# A Systematic Review of *Listeria* Species and *Listeria monocytogenes* Prevalence, Persistence, and Diversity throughout the Fresh Produce Supply Chain

**DOI:** 10.3390/foods10061427

**Published:** 2021-06-20

**Authors:** Anna Townsend, Laura K. Strawn, Benjamin J. Chapman, Laurel L. Dunn

**Affiliations:** 1 Department of Food Science and Technology, University of Georgia, 100 Cedar St., Athens, GA 30602, USA; annamtownsend@uga.edu; 2 Department of Food Science and Technology, Virginia Tech, 1230 Washington St. SW, Blacksburg, VA 24061, USA; lstrawn@vt.edu; 3 Department of Agricultural and Human Sciences, North Carolina State University, Campus Box 7606, Raleigh, NC 27695, USA; bjchapma@ncsu.edu

**Keywords:** food safety, environment, detection, identification, foodborne pathogen, contamination

## Abstract

*Listeria monocytogenes* is an increasing food safety concern throughout the produce supply chain as it has been linked to produce associated outbreaks and recalls. To our knowledge, this is the first systematic literature review to investigate *Listeria* species and *L. monocytogenes* prevalence, persistence, and diversity at each stage along the supply chain. This review identified 64 articles of 4863 candidate articles obtained from four Boolean search queries in six databases. Included studies examined naturally detected/isolated *Listeria* species and *L. monocytogenes* in fresh produce-related environments, and/or from past fresh produce associated outbreaks or from produce directly. *Listeria* species and *L. monocytogenes* were detected in each stage of the fresh produce supply chain. The greatest prevalence of *Listeria* species was observed in natural environments and outdoor production, with prevalence generally decreasing with each progression of the supply chain (e.g., packinghouse to distribution to retail). *L. monocytogenes* prevalence ranged from 61.1% to not detected (0.00%) across the entire supply chain for included studies. *Listeria* persistence and diversity were also investigated more in natural, production, and processing environments, compared to other supply chain environments (e.g., retail). Data gaps were identified for future produce safety research, for example, in the transportation and distribution center environment.

## 1. Introduction

The genus *Listeria* contains 17 species, but *Listeria monocytogenes* (*Lm*) is the only known pathogenic strain [1]. *Lm* is a bacterial, intracellular parasite that causes approximately 1600 illnesses and 260 deaths annually within the United States [2]. Regulation from the U.S. Food and Drug Administration (FDA) has a zero tolerance for *Lm* in food due to the high mortality rate associated with listeriosis, and its undetermined infectious dose [3,4]. *Lm* is able to penetrate the blood-brain and placental barriers, increasing severity of disease, compared to other foodborne pathogens that predominantly infect the gastrointestinal tract [5]. Pregnant women, elderly, infants, and immunocompromised individuals are the most at risk populations for *Listeria* infections and complications [3]. 

*Lm* has historically been a concern in ready-to-eat (RTE) foods and soft cheeses, due largely to its ability to survive and grow at refrigeration temperatures, low pH, and high salinity. *Lm* can outcompete other organisms under these less hospitable conditions [6], and has resulted in outbreaks linked to hard-boiled eggs, deli meats, raw milk, and ice cream [7]. Fresh produce is increasingly being recognized as a potential vehicle for *Lm* contamination as it often does not undergo a complete microbial kill step (i.e., often consumed raw). Mitigation of *Lm* on fresh produce can be challenging as *Lm* is naturally found and survives in soils [8,9]. Additionally, *Lm* can adhere to surfaces and form biofilms, which allow *Lm* to be resistant to desiccation, acid, heat, and/or sanitizers/disinfectants [10,11]. The combination of *Lm*’s ability to tolerate a wide array of environmental conditions, combined with its relatively high mortality rate, make *Lm* a concern throughout each stage of the produce supply chain (e.g., production, retail).

There have been at least ten recognized outbreaks of listeriosis related to fresh produce, sprouts, and mushrooms since 1979 (Table 1). *Lm* has been linked to fresh produce-associated outbreaks from products including caramel apples, cantaloupes, stone fruits, and minimally processed vegetables [12,13,14,15,16,17]. Notably, one of the most severe listeriosis outbreaks in the U.S. was from contaminated whole cantaloupe [13,14]. This outbreak led to 147 cases across 28 states with 143 hospitalizations and 33 deaths, making it the deadliest produce-related foodborne illness outbreak in the U.S. [13,14]. Thus, the goal of this systematic literature review was to synthesize the published data on *Lm* and *Listeria* spp. prevalence, persistence, and diversity throughout each stage of the fresh produce supply chain. Objectives include (1) identifying sites and associated factors (e.g., meteorological, geographical) of greatest *Listeria* and *Lm* prevalence, (2) further clarifying the relationship between *Listeria* spp. as index organisms for *Lm,* and (3) determining research needs regarding fresh produce supply chain stages.

## 2. Materials and Methods

### 2.1. Definitions and Scope

The broader genus of *Listeria* was included in this review as *Listeria* spp. are often used as index organisms for *Lm* [25]. *Listeria* spp. are defined as those species excluding *Lm,* with *Lm* reported separately unless otherwise noted. Here, in the reported review, we considered prevalence to be the occurrence of culturable *Listeria* in the natural or farm environment (e.g., soil, water), on non-food contact surfaces (NFCS) or food contact surfaces (FCS), or on fresh produce. Persistence was classified as prolonged or repeated detection of *Listeria* in an environment, or on fomites or fresh produce. Diversity was defined as species abundance and or genetic difference(s) within the *Listeria* genus and between species of *Listeria*. This review presents a panoramic view of the prevalence, persistence, and diversity of *Listeria* spp. and *Lm* throughout each stage of the produce supply chain. Stages of the produce supply chain were partitioned into seven main stages: (1) natural and outdoor production (e.g., farm), (2) packinghouse, indoor production, and processing, (3) transportation and distribution, (4) retail, (5) farmers’ market, (6) restaurant, and (7) domestic.

### 2.2. Literature Search

Searches were completed using Web of Science (beginning 1900), PubMed (beginning 1966), Food Science Source (beginning in 1941), PubAg (beginning in 1895), AGRICOLA (beginning in the 15th century), and CAB Abstracts and CAB Archives (beginning in 1973) through August 2020. Four separate Boolean searches were used: (1) *Listeria* AND prevalen* AND presen*, (2) *Listeria* AND persist*, (3) *Listeria* AND divers*, and (4) *Listeria* AND fresh AND produce. Boolean terminology was used for selected second and third terms to encompass truncations of each word. For example, “prevalen*” includes prevalence and prevalent. Searches were also restricted to English only and studies originating from U.S. based institutions or study locations. Book chapters, conference materials, and reports were excluded. 

### 2.3. Study Selection

**Figure 1:**
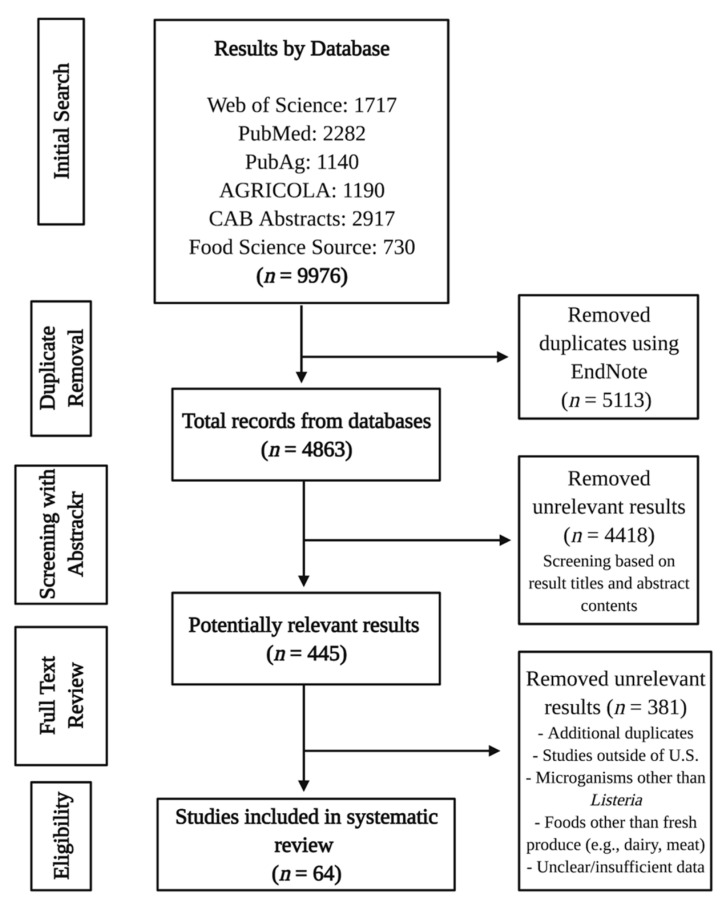
Flow diagram illustrating the systematic literature review process.

The study selection process was conducted by co-author Townsend. Results from database searches were exported to EndNote (Version X9, Clarivate, Philadelphia, PA, USA). Duplicate results were removed in EndNote using the “Find Duplicates” function after importing search results from each individual database. The remaining results were screened for relevance using a free, open-source web tool Abstrackr [26], which maintains a digital paper trail on screening decisions. Abstracts were rejected if they did not contain specified review terms (e.g., “*Listeria*,” “produce,” “fresh,” “fruit,” “prevalen”). Screening results from Abstrackr were downloaded as a CSV file containing the assigned Abstrackr (internal) ID, source ID, PubMed ID, keywords, abstract, title, journal, authors, tags, and screening decision for each study. Abstrackr assigned a score of -1 (rejected), 0 (unsure), or 1 (accepted) for the reviewer’s screening decisions. Studies were re-screened in Excel (Microsoft Corp., Redmond, WA, USA) to verify correct screening decisions through Abstrackr by examining titles and abstracts. Duplicates not identified through EndNote were also removed manually during re-screening. Studies receiving a score of 0 or “unsure” were reviewed using the full text if the title and abstract were not sufficient to gauge relevance. After re-screening, results were filtered to those with a score of 1 and were examined using the full text. Full text review evaluated whether the study included fresh produce or was directly related to the fresh produce supply chain stages. Exclusion characteristics included studies completed outside of the U.S. and focus on microorganisms other than *Listeria*. Experimental studies evaluating isolation and/or detection methods were not included if samples did not contain naturally occurring *Listeria* species from produce-related environments or *Listeria* isolates from fresh produce or from previous fresh produce-associated outbreaks. An overview of the systematic review process can be seen in Figure 1. 

### 2.4. Data Extraction and Synthesis

Data extraction and synthesis were completed by co-author Townsend. Studies were assessed individually, grouped by supply chain stage, and discussed by U.S. Department of Agriculture (USDA) Agriculture Research Service (ARS) region and chronological order. USDA ARS regions were chosen because of their distinction between climate and geography, and available natural resources and agricultural commodities and products within each region. Data regarding the study location(s), sampling site(s), sample types, duration, organisms of interest (*Listeria* spp. and/or *Lm*), prevalence (e.g., number of *Listeria* positive samples over total samples), and strain/serotype identification were extracted from the full texts. These data were entered manually into Excel. During full text review, studies were flagged for discussion if they contained data or evidence on *Listeria* diversity (e.g., strain/serotype data) and persistence (e.g., longitudinal studies). 

## 3. Results

### 3.1. Included Study Characteristics

The initial primary search identified 9976 results. After duplicate removal, 4863 studies remained for screening. After analyzing result titles and abstracts in Abstrackr, 4418 results were removed, leaving 445 results for selection criteria evaluation. Sixty-four studies were considered relevant for review based on criteria. Of the 64 studies, 54 included prevalence data for *Listeria* spp. and *Lm* based on detection of these microorganisms from environmental samples. Included studies examined a broad variety of sample types. Sample types from natural and outdoor production environments included surface water (non-irrigation and irrigation), soil, compost (raw and treated), feces (wild and domestic animals), fresh produce, and drag swabs, as well as man-made materials, such as sidewalks and doors, in urban environments. Sample types from packinghouse, indoor production, and processing environments included NFCS and produce (e.g., leafy greens, stone fruits). All retail and farmers’ market environments included produce, such as leafy greens, sprouts, packaged salads, and mushrooms, with one study also examining NFCS and FCS in retail grocery departments. The only domestic-related study examined NFCS and FCS (e.g., refrigerators, sinks, dishcloths) in consumer homes. Most identified studies performed a microbial survey of various fresh produce-related locations, while a handful of studies examined methods validation, and modeling.

Relevant studies were organized by fresh produce supply chain and then by geographic region. Geographic regions approximately follow the USDA Agricultural Research Service (ARS) regions (Northeast, Southeast, Midwest, Plains, and Pacific West) with some deviation due to multiregional studies or studies with unspecified locations. Studies with sampling locations in states or areas within two or more geographical regions were classified as multiregional. Studies not pertaining to a specific geographic region, such as laboratory-based experiments, were included in a separate “Additional Relevant Studies” section. Summary information from identified studies that assessed the prevalence of *Listeria* spp. and *Lm* throughout the fresh produce supply chain are provided in Table 2. In Table 2, those studies with only *Listeria* spp. as target organisms examined all *Listeria* species prevalence (including *Lm*). However, those studies with *Listeria* spp. and *Lm* as target organisms exclude *Lm* from the *Listeria* spp. prevalence. The overall range of *Listeria* spp. and *Lm* prevalence in each supply chain stage can be found in Table 3. 

### 3.2. Natural Environment and Outdoor Production

Twenty-five studies evaluated the prevalence, persistence, and/or diversity of *Listeria* species in the natural and/or outdoor production (e.g., farm) environment. Among the included natural environment and outdoor production studies, *Listeria* spp. prevalence ranged between undetected and 46.81%, while *Lm* prevalence ranged from undetected to 61.1% (Table 3). The largest reported *Listeria* spp. prevalence (including *Lm*) was in market-ready organic composts [28], while the largest reported *Lm* prevalence was in surface water in California [31]. Three of the relevant studies [35,44,46] in natural environment and outdoor production did not detect *Listeria* spp. (including *Lm*). No relevant studies were identified in the U.S. southeastern region. 

#### 3.2.1. Northeast Studies

In 2003, different types of compostable manures in Maryland were evaluated for gastrointestinal pathogens [36]. Overall, 12.5% of manures contained *Lm*; of these, two samples were liquid dairy manure, and one sample was cow manure [36]. These manures were also applied to potato fields, and there was no detectable *Lm* on potatoes grown in the manure-applied fields [36]. 

In 2005, a study [41] characterized eighty *Lm* isolates from urban and natural environments and observed the majority of natural isolates in lineage II, while urban isolates were evenly split between lineages I and II [41]. Seven and twenty-six *Eco*RI ribotypes were also differentiated between urban and natural environments, respectively [41]. Additionally, this study examined an additional 921 isolates from farm, food and food processing facilities, and clinical samples in combination with the previous 80 isolates to determine lineage disparities between sample categories [41]. Isolates from human clinical samples were more commonly identified as lineage I, while those from the natural environment, farms, and food were more often characterized as lineage II [41]. Ribotype diversity was also highest for isolates from farms and lowest for those in natural environments; therefore, isolates from different locations appear to be diverse [41]. 

A separate study [42] examined 1805 samples (soil, water, and environment) in both natural and urban environments. While prevalence of *Listeria* spp. was similar between natural and urban environments (23.4% and 22.3%, respectively), there were statistically significant differences in the species and allelic types between natural and urban areas [42]. There was also evidence of persistent species and allelic types during the course of the study in specific sampling sites [42]. In another study [47], allelic diversities were exhibited among *Lm* isolates from soil, water, fecal, and drag swab samples from fruit and vegetable farms. Three allelic types of *sigB* (57, 58, and 61) were determined from isolates identified from water samples [47]. *Lm* isolates from this study also represented nine allelic types from lineages I, II, and IIIa [48]. Soil available water storage, temperature, proximity to water, roads and urban development, and pasture/hay grass had an effect on the likelihood of detecting *Lm* [47]. Recent cultivation of fields and application of manure were significantly associated with an increased likelihood of isolating both *Lm* and *Salmonella* from produce fields [48]. 

In 2010, a study [49] identified meteorological and landscape factors and management practices associated with prevalence of several foodborne pathogens, such as *Lm.* Terrestrial samples from recently irrigated fields had greater likelihood of *Lm* isolation [49]. In 2014, prevalence and diversity of *Listeria* spp. were determined from environmental samples from produce production and natural environments [25]. Additionally, detection of *Listeria* was associated with identified geographical and meteorological factors [25] Random forest models suggested that soil moisture and proximity to water and pastures were highly associated with *Listeria* spp. isolation from produce production [25]. Alternatively, elevation, study site, and proximity to pastures were highly associated with *Listeria* spp. isolation from natural environments [25]. In 2015, a study examining spatial and temporal factors associated with *Lm* prevalence in spinach fields also found that *Listeria* spp. and *Lm* isolates that were associated with irrigation events showed significantly lower *sigB* allele type diversity than those isolates associated with rain events [40]. Based on this result, it was suggested that irrigation water may be a source of contamination as isolates were more similar to one another [40].

A study examining meteorological risk factors associated with a mixed produce and dairy farm in Maryland assessed 159 samples of various animal-related and environmental samples (e.g., cow feces, cow drinking water, cow and bird feces, surface water, partially and fully composted material, water, and soil from vegetable production area) over 14 months [37]. *Listeria* spp. were found in 5.03% of samples, while *Lm* isolates were obtained from 1.26% of samples [37]. 

Surface water from a non-tidal freshwater creek in Maryland was evaluated for bacterial pathogens and applied to produce fields containing kale and radishes to evaluate pathogen prevalence in soil and produce [39]. *Lm* was detected in and enumerated from creek water at 0.04 and 0.07 MPN/L, but prevalence regarding total sample number was not specified [39]. Conventional water sources, including tidal freshwater rivers, non-tidal freshwater creeks, reclaimed water holding ponds, pond water sites, and produce wash water in the mid-Atlantic U.S. were examined for *Lm* prevalence [45]. *Lm* was found in 31.18% of samples in all conventional water sources [45]. 

#### 3.2.2. Midwest Studies

Various experimental treatments were applied to varying compost amendments (sawdust, straw, and water) to monitor the change in pathogen prevalence during simulated composting [34]. Compost was sampled on days 0, 3, 7, 14, 28, and 56 for pathogen presence, and *Listeria* species (including *Lm*) were found in all three treated compost types on days 0 and 3; however, their prevalence was not reported [34]. 

#### 3.2.3. Plains Studies

In 2002, a study [38] examining cabbage farms and packinghouses in the Rio Grande Valley and Texas found 2.81% *Listeria* spp. and 3.04% *Lm* prevalence among cabbage, water, and environmental samples. 

A study performed in five Colorado pristine wilderness areas detected *Listeria* spp. in all tested sample types (soil, water, sediment, surface soil, and wildlife feces) and *Lm* from only water and feces [27]. Additionally, this study observed approximately 75% of *Listeria* spp. positive samples from a single wilderness location [27]. It is suggested that some *Lm* strains may be persistent, as one unique *Lm* strain was found in both the summer of the first sampling year and in the fall of the second sampling year [27]. Overall, this study indicated that *Listeria* incidence was rare at the study site and isolates tended to be genetically distinct based on wilderness area [27]. 

#### 3.2.4. Pacific West Studies

Four separate studies in California evaluated the microbial composition of watershed areas and all found *Lm* in surface water samples [30,31,32,33]. *Lm* prevalence ranged from 30.1 to 61.1% across the four studies. Two studies [30,32] found a range of serotypes, 1/2a, 1/2b, 3a, 4b, 4d, and 4e, indicating strain-level diversity in and among watershed sites in California. One study [79] characterized over 100 *Lm* isolates from watersheds near leafy green production sites using MLVA and found 49 different *Lm* strains, illustrating the diversity of strains within these sites. In Arizona, *Lm* was not detected in reclaimed and return flow water in two wastewater treatment plants [44]. 

One study examined market-ready organic compost from 94 compost facilities across Washington, Oregon, and California and detected a *Listeria* species prevalence of 46.81% [28]. Another study did not detect *Lm* in fertilizer, soil, foliar, and raspberry fruit from raspberry fields in Washington [46].

#### 3.2.5. Multiregional Studies

Two studies contain sampling regions across U.S. geographic regions. A study over nine states (AZ, CA, GA, KY, MD, NY, NC, SC, and TN) examined over 100 organic fertilizers (e.g., spent-mushroom compost, vermicompost, mixed animal waste) and did not detect *Lm* [29]. Another multiregional study [43] examined interactions between weather and microbial and physiochemical water quality to determine the likelihood of pathogens in agricultural water in Arizona and New York. Two types of sampling methods were used: Moore swabs (MS), which use sterile gauze or cheesecloth to continuously filter microorganisms from water, and grab swabs (GS), which collect a single volume of water for analysis [43]. The odds of isolating *Lm* using MS was significantly lower than that of isolating *Lm* from paired GS [43]. However, more competitive microflora and fewer *Listeria*-like colonies were observed after plating MS enrichments compared to GS enrichments [43]. Highly ranked factors associated with *Lm* isolation from both MS and GS included flow rate of agricultural water, weather conditions 0–4 days and 0–1 day before sample collection [43]. 

### 3.3. Packinghouse, Indoor Production, and Processing

Eighteen identified studies included microbial sampling at indoor production, packinghouse, or processing environments. These studies primarily focused on non-food contact surface areas, while some studies performed microbiological analysis of produce. Five studies evaluated environmental and some produce samples from packinghouses. 

#### 3.3.1. Northeast Studies

Two studies focused on mushroom production and processing facilities. The first sampled NFCS in a small-scale mushroom production facility in Pennsylvania and detected *Listeria* spp. and *Lm* in 14.13% and 1.63% of all samples [50]. *Listeria* species positive samples included phase I composting, phase II composting, tray filling line, and growing rooms, while *Lm* positive samples were only found in phase I composting [50]. Identified *Listeria* species other than *Lm* included *L. innocua, L. welshimeri,* and *L. grayi* [50]. The second study examined NFCS in a fresh mushroom slicing and packaging operation [60]. The location of the packaging facility is not specified in this study; however, based on the sampling methodology it is reasonable to assume the facility is in Pennsylvania. *Listeria* species were found in 6.27% of samples, where positive samples included those in receiving and staging, washing and slicing, packaging, and shipping sites [60]. *Lm* was detected in 18.8% of samples, with positive samples in receiving and staging, washing and slicing, and packaging sites [60]. 

NFCS samples were taken at apple and other tree fruit packinghouses in the northeastern U.S. [53]. *Lm* was found in 56.41% of NFCS samples [53]. Positive samples included washing drying, and waxing areas of the packinghouse facilities [53].

One northeast-based studies failed to detect any *Listeria* spp. or *Lm* in produce samples. *Lm* was not detected in tomatoes, leafy greens, peppers, cucumbers, and other produce (*n* = 177) from seven organic farms in Maryland [51]. Well water and surface water (*n* = 29) were also tested at these organic farms and *Lm* was not detected [51].

#### 3.3.2. Southern/Southeastern Studies

The microbiological quality of leafy greens, herbs, and cantaloupes was evaluated, and *Lm* was detected in three cabbage samples—6.98% prevalence out of all samples (*n* = 43) [54]. Another study examining leafy greens, herbs, and cantaloupe from thirteen farms and five packing sheds in the southern U.S. did not detect any *Lm* [52].

A study based in the southeastern U.S. evaluated environmental samples collected from eleven fresh produce packinghouses and found 2.64% of samples were positive for *Listeria* species and 3.15% for *Lm* [55]. Positive samples included drains, cold storage rooms, wet NFCS, mobile NFCS, dry NFCS, and outside packing/handling areas [55]. Non-*Lm Listeria* species isolated included *L. innocua*, *L. marthii, L. seeligeri*, and *L. welshimeri* [55].

#### 3.3.3. Pacific West Studies

In the Pacific northwest U.S., seven produce handling and processing facilities were environmentally sampled for both *Listeria* spp. and *Lm* [58]. The detectable *Lm* strains represented serotypes 1/2a, 3a, 4b, 4d, and 4e [58]. In addition to *Lm,* researchers also detected *L. innocua, L. ivanovii,* and *L. welshimeri* [58]. Positive sample locations included drains, entry points, floors, forklift tires, and equipment legs [58]. 

Stone fruits, such as white and yellow nectarines and white and yellow peaches, were acquired from a packinghouse environment in California from lots associated with the 2014 stone fruit-associated listeriosis outbreak [17]. Implicated stone fruits from were examined for listeria prevalence, and 53.3% of all samples were positive for *Lm* [17]. Of all nectarines sampled, 25% were positive for *Lm* and of all peaches, 91.1% were positive for *Lm.* Serotypes of *Lm* were determined to be IVb-v1 and 1/2b [17]. SNP-based whole-genome sequencing demonstrated the outbreak-associated isolates differed by up to 42 SNPs compared to unrelated clinical isolates [17]. Isolates belonging to serotype Ivb-v1 belonged to the singleton ST382, which is an emerging clonal group of *Lm* and was associated with the 2011 cantaloupe-related outbreak [17]. However, a core genome MLST study to identify globally distributed clonal groups and outbreak strains of *Lm* did not associate ST382 with the cantaloupe-associated outbreak [80]. ST382 was only associated with caramel apple, stone fruit, and packaged leafy green salad outbreaks [80]. Otherwise, both studies emphasize ST382 as an emerging clonal group associated with produce [17,80]. 

Lastly, an analysis of microflora on organically and conventionally grown spring mix from a California processor did not yield any *Lm* isolates [59].

#### 3.3.4. Multiregional Studies

Baby spinach samples from farms in TX, AZ, CA, CO, MD, and NJ and Ontario and Quebec, Canada were examined from two processing plants (unspecified location) for *Listeria* spp. and *Lm* [57]. *Lm* was detected in one processed and two minimally processed spinach samples (3/409; 0.73%), while *L. seeligeri* was found in two processed spinach samples (2/409; 0.49%) [57].

A study in three packinghouses and five fresh-cut facilities on both the East and West coasts of the U.S. environmentally sampled zones 2 and 3 for *Listeria.* Zone 2 consisted of areas that are in close proximity to food or FCS, while zone 3 included areas in the processing and packing area that are not adjacent to FCS [56]. *Listeria* spp. and *Lm* were found in zones 2 and 3 in packinghouses and only in zone 3 for fresh-cut facilities [56]. 

#### 3.3.5. Other Relevant Studies

In 2017, researchers examined the genetic relatedness of 387 *Lm* serotype 4b strains, with some strains previously isolated from caramel apples, lettuce, collard greens, curly parsley, nectarines, peaches, spinach, and walnuts [81]. While there were clinical and non-clinical samples in each of the seven clades identified by the CFSAN SNP Pipeline, nearly all of the strains were organized into one clade by the multi-virulence-locus sequencing type (MVLST) analysis [81]. These findings suggest that 4bV strains may be under different selection pressure at varied geographic regions, which may conserve diversity across virulence loci but permit variability across less highly conserved areas of the genome [81]. 

From a diversity perspective, copper stress in *Lm* isolates and mutants from the 2011 cantaloupe-associated outbreak can be mediated by the penicillin-bind protein, *pbp4* [82]. A transposon mutant of *pbp4* demonstrated reduced copper tolerance and minimal inhibitory concentrations for selected cell wall-active antibiotics [82]. Genomic characterization of the cantaloupe-associated outbreak strains found that 1/2b isolates were closely related, while 1/2a isolates comprised two genomic groups [83]. An analysis of non-hemolytic mutants of a 1/2b strain that contributed to the cantaloupe-associated outbreak illustrated that *prfA* and *hly* contribute to biofilm formation and aggregation of *Lm* [84]. *PrfA* and *hyl* are well-known *Lm* virulence genes [41]. 

Genetic diversity of *Listeria* isolates from food production facilities, including farms and packinghouses, was investigated to assess the relationship among isolates [85]. In addition to whole genome sequencing (WGS) data, metadata from the FDA Field Accomplishments and Compliance Tracking System (FACTS) database was also included in the analysis to link isolates to facilities [85]. SNP data indicated that isolates from different facilities, as well as isolates within the same facility, have fairly large genetic distances (>20 SNPs) [85]. 

### 3.4. Transportation and Distribution

There were no studies identified that investigated *Listeria* spp. and *Lm* presence, persistence, and/or diversity in regard to fresh produce transport and distribution. 

### 3.5. Retail

Fifteen relevant studies involve microbiological analysis of NFCS, FCS, or fresh produce acquired in retail environments. Only one study examined FCS and NFCS [64], while all other identified studies microbially examined produce from retail locations. The range of *Listeria* prevalence at the retail level was 6.13 to 10.71, while that of *Lm* was not detected to 4.42% (Table 3). 

#### 3.5.1. Northeast Studies

In Delaware, mushrooms and alfalfa sprouts (*n* = 408) were acquired from grocery stores over a period of 15 months [68]. *Listeria* spp. were more frequently detected in mushrooms (8.42%) than sprouts (3.88%), while *Lm* was only detected in sprouts (0.49%) [68].

One study that investigated food safety risk differences in RTE fresh fruit, greens, and herbs acquired from retail locations in Philadelphia, PA did not detect *Lm* [71].

Basil, cilantro, lettuce, scallions, spinach, and parsley from three retail chain groceries in Maryland were sampled bi-weekly for *Lm* presence [63]. Only spinach samples contained *Lm*, but the number of positive samples was not reported [63].

A third study out of Virginia sampled whole and sliced shiitake mushrooms purchased from retail markets for both *Listeria* spp. and *Lm* [67]. *Listeria* spp. were only found on whole mushrooms (5%), while *Lm* was not detected [67]. 

#### 3.5.2. Midwest Studies

One study examining both fresh (lettuce, potato peels, corn husks, broccoli stems, cabbage outer leaves, carrot peels, cauliflower stems, mushroom stems, spinach, and beet peels) and frozen (green beans, pea pods, green peas, and spinach) produce from a supermarket in Minnesota did not detect *Lm* [72]. Broccoli, cabbage, carrots, cauliflower, cucumbers, lettuce, mushrooms, potatoes, radishes, and tomatoes were sampled from two supermarkets and assessed for *Listeria* spp. [73]. *Listeria* spp. were isolated from 9.7% of samples, including lettuce, cabbage, cucumbers, mushrooms, potatoes, and radishes [73]. 

#### 3.5.3. Pacific West Studies

In Washington state, a study [69] examined sprouts and mushrooms from retail stores in Seattle for the presence of *Lm*. *Lm* was not detected in any sprout samples but was detected in 1% of mushroom samples [69]. 

#### 3.5.4. Multiregional Studies

A 2007 study characterizing *Lm* strains isolated from retails foods in Florida and the greater Washington, D.C. area identified three isolates from conventionally grown fresh produce and two from organically grown fresh produce [86]. Serotype designations for these isolates are not explicitly stated, but may include 1/2b, 1/2c, 3b, 3c, 4a, or 4c [86]. Isolates from conventional produce were all resistant to sulfonamide, while those from organic produce were resistant to either ciprofloxacin or sulfonamide [86]. 

Fresh cut vegetables, low-acid fruit, and sprouts were acquired from 1,042 retail stores across California, Maryland, Connecticut, and Georgia [66]. *Lm* was detected in 0.53% (36/6,749) of samples [66]. Another multistate investigation of fresh produce from retail locations over six years found *Lm* in leafy greens (0.11%), sprouts (0.11%), and melons (0.23%) [70]. 

In Michigan and New Jersey, several produce items were sampled from the farm (cucumbers), prior to retail distribution (cilantro), and in retail (cilantro and mung bean sprouts) to evaluate their microbiomes [61]. *Listeria* spp. and *Lm* isolates were not obtained; however, species level proportional abundances identified *Listeria* DNA in two cilantro samples [61]. 

A systematic review of the prevalence of *Listeria* in RTE foods incorporated retail-acquired produce from South America, North America, Europe, Africa, and Asia [62]. Packaged salad samples had a *Lm* prevalence of 2.60% [62]. Another study [65] analyzed approximately 3000 bagged salad samples in Maryland and California and isolated *Lm* from 0.75% of samples. 

Thirty retail grocery produce departments across CA, TX, IA, MN, OH, MA, and FL were environmentally sampled for *Lm* [64]. Positive sample types included areas such as cold room storage drains, standing water, produce area drains, squeegee/floor cleaners, and the cold room storage floor [64]. *Lm* was isolated from 4.42% of all samples (both FCS and NFCS) [64].

#### 3.5.5. Other Relevant Studies

Heavy metal and disinfectant resistance were assessed in *Lm* strains from foods and food processing plants with unspecified locations [87]. Isolates from fresh avocados and other avocado products (e.g., paste, pulp, chunks) demonstrated little resistance to heavy metals (cadmium, benzalkonium chloride, and arsenic) [87]. Only two 4b serotypes, one from avocado and the other from frozen avocado pulps, demonstrated resistance to cadmium and arsenic [87]. None of the avocado and processed avocado isolates contained cadmium resistance determinants (*cadA1, cadA2, and cadA3*) [87]. 

### 3.6. Farmers’ Market

Five studies examined produce from farmers’ markets for *Listeria*. The range of *Listeria* spp. prevalence was not detected to approximately 15.0%, while that of *Lm* was not detected to 4.72%. No *Lm* isolate serovars were identified in relevant studies at the farmers’ market level.

#### 3.6.1. Northeast Studies

A variety of produce was sampled from both farmers’ markets and supermarkets in Washington, D.C. [76]. *Listeria* spp., including *L. innocua, L. welshimeri,* and *L. grayi*, were isolated from celery, field cress, lettuce, mung bean sprouts, potatoes, soybean sprouts, watercress, and yams [76]. *Lm* was isolated from field cress and potatoes [76]. This study observed the greatest *Listeria* spp. and *Lm* prevalence at the farmers’ market level compared to that from the other relevant farmers’ market studies (Table 3). Additionally, this study isolated *Listeria* from produce acquired from both supermarkets and farmers’ markets [76]. *Listeria* positive samples obtained from farmer’s markets included celery, field cress, and potatoes, while those from supermarkets were celery, lettuce, mung bean sprouts, soybean sprouts, watercress, and yams [76]. 

Leafy greens (lettuce, spinach, and kale) were acquired from 58 vendors at 25 farmers’ markets in Pennsylvania [75]. Both *Listeria* spp. and *Lm* were isolated from kale, lettuce, and spinach, and spinach, respectively [75].

#### 3.6.2. Southeast Studies

In Florida, leafy greens, berries, spinach, and tomatoes acquired from nine farmers’ markets and twelve supermarkets were evaluated for *Lm* [77]. *Lm* was found on leafy greens and spinach from farmers’ markets; no *Lm* was detected in any supermarket-acquired produce [77].

#### 3.6.3. Multiregional Studies

*Listeria* spp. and *Lm* were recovered from produce collected at two farmers’ markets, one of which was in Kentucky and the other in West Virginia [74]. *Listeria* spp. were isolated from peppers and cantaloupes, while *Lm* was isolated from tomatoes, cucumbers, and cantaloupes [74]. Additionally, the research group investigated farmers’ market vendors’ handling of produce containers and the survival of *Lm* on plastic, pressed card, and wood container surfaces [88]. Two *Lm* isolates with serotype 1/2b from the cantaloupe-associated outbreak were used for inoculation of container surfaces [88]. The initial population of *Lm* recovered from each material surface after inoculation ranged from 6.39 to 6.93 log CFU/cm^2^ at 3.2 and 22.5 °C, respectively [88]. After the first day of storage, *Lm* populations were fairly constant or only slightly decreased [88]. By day 21, the *Lm* population remaining on all surfaces ranged from 4.89 to 5.73 log CFU/cm^2^ [88]. *Lm* persisted longer and at a greater population level on pressed card surfaces compared to on plastic and wood surfaces [88]. 

### 3.7. Restaurant

There were no relevant studies that investigated *Listeria* spp. and *Lm* prevalence, persistence, and/or diversity in restaurant environments.

### 3.8. Domestic

One relevant study was identified examining consumer kitchens and homes. One hundred homes in Philadelphia, PA were sampled over one year for *Listeria* spp. and *Lm* [78]. Types of samples included kitchen areas, such as refrigerator door handles and interiors, counters, and used sponges and dishcloths [78]. *Listeria* spp. were found in the meat drawer of refrigerators, which accounted for 2.15% (12/557) of samples [78]. *Lm* was present on refrigerator door handles, refrigerator drawers, kitchen sinks, and on dishcloths/sponges, which accounted for 0.72% (4/557) of samples [78]. 

## 4. Discussion

### 4.1. Natural Environments and Outdoor Production

Detection of *Listeria* spp. and *Lm* from natural environments and outdoor production provides insight into identifying potential sources and routes for cross-contamination onto produce. The extent of *Listeria* prevalence and persistence may also depend on the environment. The ubiquity of *Listeria* in natural environments presents a baseline level of risk for fresh produce at the farm level. Persistence of *Listeria* in natural or farm environments is especially challenging as harborage sites can be difficult to determine, access, or transplanted from other sources. Therefore, *Listeria* prevention at the farm level will remain a concern for producers, produce handlers, and processors. In 2016, the Produce Safety Rule (PSR) within the Food Safety Modernization Act was finalized and established science-based standards for the safe production of fresh produce for human consumption. Based on the identified studies focusing on *Listeria* prevalence at the natural environment and outdoor production level, surface water samples have a consistently high percentage of *Listeria*-positive samples. Quality and safety of surface water should be prioritized, as it is under the PSR, to mitigate possible cross-contamination of *Listeria* onto fresh produce. Furthermore, natural events or production activities including irrigation, precipitation, and other on-farm management practices or inputs impact *Listeria* isolation; these events can be observed and when possible, modified to limit the prevalence of *Listeria* in natural and farm environments [25,40,43,47,48,49]. As *Listeria* has demonstrated to be prevalent, persistent, and diverse at the natural environment and outdoor production level, it is crucial for producers to adhere to Good Agricultural Practices (GAPs) and Good Handling Practices (GHPs) to prevent cross-contamination of *Listeria* onto produce from agricultural water, soil, workers, and equipment during harvest and packaging. Research areas of need include those completed in the southeast region, as this review did not identify any relevant studies in that region. 

These studies also support molecular source tracking and fingerprinting of *Listeria* species that naturally occur in the environment, which is useful for foodborne illness outbreak traceback investigations and evidence of persistent species or strains [42]. The longitudinal study data reported in this review also suggests persistence of strains within certain environments. A range of *Listeria* diversity was exhibited in relevant studies at the natural environment and outdoor production level, with ten studies identifying *Listeria* species other than *Lm* and/or serotyping *Lm* strains [25,27,30,32,38,42,43,48,49,50]. Therefore, not only may certain *Listeria* species be persistent in natural and farm environments, but they may also be diverse. This evidence also supports the use of *Listeria* spp. as index organisms for *Lm*, as many studies at this supply chain stage isolated both *Listeria* spp. and *Lm* from the same sample types and locations. Generally, these studies found a greater prevalence of *Listeria* spp. compared to *Lm,* which is a criterion of an acceptable index organism [25]. However, the *Listeria* spp. isolated at this supply chain stage include both *Listeria sensu stricto* (i.e., isolated from animal hosts), as well as *Listeria sensu lato* (i.e., inability to colonize animal hosts) [89]. Therefore, it may not be appropriate to classify certain *Listeria* spp. as index organisms for *Lm* due to differences in metabolic activity and pathogenicity. This is consistent with conclusions in Chapin et al. (2014), where the strength of association between isolation of *Listeria* spp. and *Lm* may depend on the *Listeria* species.

### 4.2. Packinghouse, Indoor Production, and Processing

Across these studies, both *Listeria* spp. and *Lm* were found in all food contact zones (i.e., ranging from food contact surfaces to remote areas of a facility). There was also evidence that some *Lm* strains are from the same source or are persistent in this environment [50]. Contamination within several facility zones may indicate movement of personnel and/or equipment between zones that could contribute to contamination [50]. Therefore, limitation of movement between zones and review of cleaning and sanitation operations may reduce *Listeria* prevalence throughout the facility [50]. Cleaning and sanitation operations within processing facilities should focus on specific surfaces (e.g., porous, difficult to clean) that may offer greater harborage or support of *Listeria* populations. 

One identified study investigated strain-level diversity of *Listeria* in stone fruit in response to the 2014 stone fruit-associated listeriosis outbreak [17]. This outbreak is an example of a polyclonal outbreak where several strains of the bacterial pathogen are linked to the outbreak. Polyclonal outbreaks may be indicative of single or multiple persistent *Listeria* strains that may establish in a facility and undergo selection over time, or strains that may transplant to other locations [90]. Strain diversity can complicate traceback investigations by increasing the difficulty of matching clinical isolates with implicated food isolates. Incidence of polyclonal outbreaks has further bolstered the necessity of WGS for molecular surveillance and fingerprinting of foodborne pathogens [90]. Therefore, more studies are needed that focus on strain-level identification and fingerprinting of *Listeria* species at this supply chain stage. 

*Listeria* is also prevalent and diverse in packinghouse and processing environments; however, more research is needed to address persistence of strains within these environments. Attention should also be placed toward relevant environments in the Midwest and plains regions, as no relevant studies were identified in those areas for this review. While some studies suggest persistence of isolates, another suggests a reintroduction of *Listeria* species within produce packinghouses, rather than persistence [55]. Therefore, it is critical for studies to sample locations frequently and use high-resolution subtyping to more accurately assess strain persistence. Verifying that cleaning and sanitation procedures are preventing establishment and colonization of *Listeria* will assist in minimizing *Listeria* prevalence and possible persistence or reintroduction within a facility.

### 4.3. Transportation and Distribution

Transportation vehicles and distribution centers can be bottlenecks within the food supply chain. Within this supply chain stage, large quantities and varieties of products move into a singular location and are then dispersed to many retail, foodservice, or secondary storage areas. As the majority of products in transport and distribution are in their final packaging and typically in secondary or even tertiary containers, they are considered to be at low risk of contamination. Therefore, few best practices are implemented with food safety or current Good Manufacturing Practices in mind. Additionally, as most products sold at major grocers, restaurants, or food service facilities pass through at least one distribution center before reaching the consumer, it is reasonable to assume that many foods associated with foodborne illness outbreaks transit through this type of facility. Without data identifying the hazards associated with environmental contamination in this supply chain stage and quantifying the likelihood for microbial hazards to penetrate and subsequently contaminate fresh produce in unsealed containers, it is difficult to assess the degree of risk associated with transit of these products. Because no studies were identified that assess *Listeria* spp. and *Lm* prevalence, persistence, and diversity at the transportation and distribution stage, research is needed in this area. 

### 4.4. Retail

Many retail studies focusing on *Listeria* have occurred in delicatessens, as RTE salads and deli meats are foods traditionally associated with *Listeria* contamination. Additional studies on *Listeria* prevalence, and especially regarding persistence and diversity, are needed in retail produce departments. This review did not identify any relevant studies in the southeastern and plains regions either. Nearly all (14/17, 82.4%) identified retail-based studies, with publications ranging between 1988 and 2020, detected *Listeria* and/or *Lm* in fresh produce samples. Therefore, *Listeria* has been found in retail fresh produce prior to and after the implementation of the “zero-tolerance” policy. This is an indication that *Listeria* is a consistent issue in retail fresh produce with contributing factors not yet identified or addressed through food safety regulations. RTE foods, including many types of fresh produce, do not undergo additional “kill steps,” such as cooking, so it is critical that no opportunity exists for retail produce items to become contaminated with foodborne pathogens. Frequent and thorough cleaning and sanitation of retail produce departments, as well as good handling practices, should be implemented to avoid cross-contamination and establishment of *Listeria* species and other pathogenic microorganisms. 

While many relevant retail-based studies occurred over long durations of time, these studies did not characterize *Listeria* or *Lm* isolates at the species or strain level. Therefore, persistence of specific isolates is unable to be determined. While there are legal hurdles, future research should focus on high-resolution identification of isolates from frequently sampled retail fresh produce environments to determine *Listeria* persistence. Additionally, more prevalence data between *Listeria* spp. and *Lm* is needed to designate *Listeria* spp. as index organisms at the retail level. 

### 4.5. Farmers’ Market

Supermarkets are subject to governmental regulations and have numerous resources to implement food safety practices, while farmers’ markets typically do not have strict, defined food safety guidelines. Additionally, relevant studies completed at farmers’ market locations illustrate a greater prevalence of *Listeria* compared to that of supermarkets. Farmers’ markets vendors are generally exempt from federal regulations and may have little oversight. Knowledge and implementation of food safety practices of farmers’ market vendors and managers have been previously investigated [91,92,93]. Some practices may put consumers at risk of contracting foodborne illness as some markets do not have food safety plans nor provide food safety guidance [93]. Lack of facilities, equipment, and resources with food safety guidelines can be barriers to implementing food safety practices [91]. Therefore, farmers’ market-specific training may be able to improve food safety practices, as generalized food safety trainings may not lead to behavioral changes [91,92,93]. 

Only one identified study [76] characterized the diversity of *Listeria* species in farmers’ market produce. Therefore, future studies should also examine the genetic diversity of *Listeria* spp. in farmers’ market environments and produce. This review identified research gaps in geographic regions, where there were no relevant studies identified in the upper Midwest, plains area, and Pacific west. Additionally, more sample types focusing on NFCS are needed at the farmers’ market level, as the identified studies only focused on fresh produce sampling. Isolate characterization is also needed to characterize the diversity of *Listeria* and validate *Listeria* spp. as index organisms for *Lm* at the farmers’ market level and assist in isolate tracking from farm to market. 

### 4.6. Restaurant

While research evaluating restaurant food preparation practices and employee behaviors has been completed [94,95,96], there appear to be no studies on *Listeria* spp. and/or *Lm* prevalence, persistence, or diversity at the restaurant level. This particular research gap may not be able to be addressed due to legal implications and other reputational consequences restaurants may face if included in research studies on environmental foodborne bacteria. 

### 4.7. Domestic

Consumer behavior may lead to persistence of *Lm* and/or cross-contamination of *Lm* onto other foods. For example, poor cleaning and sanitation of home refrigerators can lead to transfer of bacteria on surfaces to foods. Improper separation of meats, soft cheeses, and other *Listeria*-associated foods and RTE foods, such as produce, can also lead to cross-contamination if *Listeria* is present. Reusable bags can also be a route of cross-contamination, especially if they are not washed often or are reused for other purposes that may pose a food safety risk [97]. Temperature abuse is a known contributor to foodborne pathogen, including *Lm*, persistence and proliferation on fresh produce [98,99,100,101,102,103]. Therefore, ensuring that foods are not held at abusive temperatures after purchase (i.e., for prolonged periods of time in a vehicle) and are properly placed in a clean, properly operating refrigerator will help prevent food safety issues. Readily available and widespread consumer-friendly food safety materials, such as internet-accessible and printed resources, may help improve food safety at home [97].

Additional studies evaluating *Listeria* spp. and *Lm* prevalence at the domestic level is needed to determine sources of *Listeria* (i.e., from the produce itself or via cross contamination). Furthermore, one relevant study does not provide enough evidence to support *Listeria* spp. as index organisms for *Lm* in the domestic environment. At this stage, *Listeria* spp. may be more appropriately considered as indicator organisms in relation to hygienic status of consumer kitchens. 

## 5. Conclusions

Based on these studies, potential *Listeria* contamination is a risk at each stage along the fresh produce supply chain. The largest prevalence of *Listeria* spp. and *Lm* was observed at the natural and outdoor production environments, with prevalence generally decreasing with each progression in the supply chain (e.g., packinghouse to distribution to retail). Generally, both *Listeria* spp. and *Lm* were isolated together in many studies at the natural environment and outdoor production, as well as at the packinghouse, indoor production, and processing stages. Therefore, *Listeria* spp. many serve as index organisms for *Lm* at these stages; however, more research is needed to address *Listeria* spp. as index organisms at subsequent supply chain stages (e.g., retail). Another research deficit includes work in the Midwest and plains regions as these areas had the fewest relevant studies. Research is also needed to examine *Lm* prevalence, diversity, and persistence in the produce transport and distribution environment. Additional strain-level studies focusing on isolates obtained from environments closer to the supply endpoint, as well as across smaller food systems (i.e., on farms that supply farmers’ markets, markets themselves, and consumer homes), will also provide evidence of *Listeria* diversity and persistence. 

## Figures and Tables

**Table 1:** U.S. listeriosis outbreaks associated with fresh produce.

Associated Food	Source of Contamination	Year	Number of States Affected	Cases	Hospitalizations	Deaths	Reference
Enoki mushrooms	TBD	2020	17	36	30	4	[18]
Frozen vegetables	Processing environment	2016	4	9	9	3	[19]
Packaged salad	Processing environment	2016	9	19	19	1	[20]
Caramel apples	Processing environment	2014	12	35	34	7	[16]
Mung bean sprouts	Production environment	2014	2	5	5	2	[21]
Peaches and nectarines	Packinghouse environment	2014	4	2	2	1	[17,22]
Cantaloupes	Processing environment	2011	28	147	143	33	[14]
Chopped celery	Processing environment	2010	1	10	10	5	[15]
Sprouts	Production environment	2008	Unknown	20	16	0	[23]
Raw celery, tomatoes, and lettuce	Unknown	1979	1	20	20	5	[23,24]

**Table 2:** Summary of *Listeria* spp. and *L. monocytogenes* prevalence throughout the fresh produce supply chain. All *Listeria* species (including *L. monocytogenes*) are considered when *Listeria* spp. are the only target organisms within a study. However, *Listeria* spp. exclude *L. monocytogenes* when both are included in target organisms within a study. Prevalence is given in number of positive samples divided by the total number of samples with corresponding percentage in parentheses.

Geographical Location(s)	Sampling Site(s)	Study Duration	Type of Sample(s)	Target Organism(s)	Presence (Yes/Not Detected)	Prevalence	Positive Sample Type(s)	Strain/Serotype	Reference
**Natural Environment and Outdoor Production**									
Colorado	Wilderness areas (*n* = 5)	Two years	Soil, water, sediment, surface soil, and wildlife feces (*n* = 572)	*Listeria* spp. (excludes *Lm*)	Yes	19/572 (3.32)	All sample types	*L. welshimeri* and undetermined strains	[27]
				*Listeria monocytogenes*	Yes	3/572 (0.52)	Feces and water	1/2a, 1/2b, 3a, 3b, or 7	
Washington, Oregon, and California	Compost facilities (*n* = 94)	Three weeks	Market-ready, organic compost (*n* = 94)	*Listeria* spp. (all)	Yes	22/47 (46.81)	Compost from OR and CA	Not identified	[28]
Arizona, California, Georgia, Kentucky, Maryland, New York, North Carolina, South Carolina, Tennessee	Farms, outside locations, commercial operations	Four years	Organic fertilizers (*n* = 103)	*Listeria monocytogenes*	ND	N/A	N/A	N/A	[29]
New York	Natural environments (*n* = 5)	Two years	Composite soil, drag swab, water, and wildlife/domestic animal feces (*n* = 1322)	*Listeria* spp. (excludes *Lm*)	Yes	186/734 (25.34)	All sample types	*L. innocua, L. seeligeri, L. welshimeri,* and *L. marthii*	[25]
				*Listeria monocytogenes*	Yes	59/734 (8.04)	Not specified	Not identified	
California	Watershed areas near produce field (*n* = 30)	Two years	Surface water (*n* = 1405)	*Listeria monocytogenes*	Yes	~ 604/1405 (43)	Surface water	1/2a, 1/2b, 3a, 4d, and 4e	[30]
California	Watershed areas near produce field (*n* = 14)	Ten months	Surface water	*Listeria monocytogenes*	Yes	Culture positive: 22/36 (61.1) PCR positive: 21/36 (58.3)	Surface water	Not identified	[31]
California	Watershed areas near produce field (*n* = 30)	Three months	Surface water (*n* = 206)	*Listeria monocytogenes*	Yes	62/206 (30.1)	Surface water	1/2a, 1/2b, and 4b	[32]
California	Watershed areas (*n* > 30)	Two years	Surface water (*n* = 860)	*Listeria monocytogenes*	Yes	381/860 (44.3)	Surface water	Not identified	[33]
Ohio	Experimental treatments (*n* = 5) with varying amendments (sawdust, straw, and water) to manure	Sampling occurred over 57-day period	Compost testing occurred on days 0, 14, 28, and 56	*Listeria* spp. (all)	Yes	Not specified	Day 0: Five compost treatments. Day 3: Three compost treatments. Day 14: One compost treatment	Not identified	[34]
Pennsylvania	Six parallel plots (5 m × 2 m)	Not specified	Irrigation water and spinach	*Listeria* spp. (all)	ND	N/A	N/A	N/A	[35]
Maine	Randomized complete block split plot design with five replications completed in duplicate	Two years	Liquid dairy, pig, chicken, cow manure, and potatoes grown with and without liquid dairy manure	*Listeria monocytogenes*	Yes	Total: 3/24 (12.5) Liquid dairy: 2/6 (33.3) Cow: 1/6 (16.7)	Liquid dairy and cow manure	N/A	[36]
Maryland	Mixed produce and dairy farm; 12 sampling sites	14 months	Cow feces, cow feed, cow drinking water, bird feces, bird gathering areas, raw liquid manure, water from lagoon, raw separated solids, partially composted material, fully composed material, surface water, and soil from vegetable production area and cow pasture (*n* = 159)	*Listeria* spp. (excludes *Lm*)	Yes	8/159 (5.03)	Cow feed, cow feces, raw separated solid, windrow compost, finished compost, bird feces, pasture soil	Not identified	[37]
				*Listeria monocytogenes*	Yes	2/159 (1.26)	Cow feed and pasture soil	Not identified	
Rio Grande Valley and Texas	Cabbage farms with packing sheds and separate packing sheds (*n* = 6)	Seven months	Cabbage (*n* = 425), water (*n* = 205), and environmental (*n* = 225)	*Listeria* spp. (excludes *Lm*)	Yes	24/855 (2.81)	All sample types	*L. grayi, L. innocua, L. ivanoii,* and *L. welshimeri*	[38]
				*Listeria monocytogenes*	Yes	26/855 (3.04)	All sample types	Not identified	
Maryland	Non-tidal freshwater creek	One year	Creek water, soil, radishes, and kale	*Listeria monocytogenes*	Yes	0.04 and 0.07 MPN/L	Creek water	Not identified	[39]
New York	Spinach fields (*n* = 2)	Seven weeks	Feces, leaves, soil, and surface water (*n* = 1492)	*Listeria* spp. (excludes *Lm)*	Yes	74/1492 (4.96)	All sample types	Not identified	[40]
				*Listeria monocytogenes*	Yes	130/1492 (8.71)		Not identified	
New York	Natural (*n* = 4) and urban (*n* = 4) sites	Two years	Soil, vegetation, surface water, floors, sidewalks, and human contact surfaces (*n* = 1805)	*Listeria monocytogenes*	Yes	Urban: 67/898 (7.5) Natural: 13/907 (1.4)	Urban: soil, vegetation, water, sidewalk/floor, and human contact surfaces Natural: soil, vegetation, and water	Not identified	[41]
New York	Natural (*n* = 4) and urban (*n* = 4) sites	Two years	Soil, vegetation, surface water, floors, sidewalks, and human contact surfaces (*n* = 1805)	*Listeria* spp. (excludes *Lm*)	Yes	362/1805 (20.1)	Variety from natural and urban sites	*L. marthii, L. innocua, L. seeligeri,* and *L. welshimeri*	[42]
				*Listeria monocytogenes*	Yes	80/1805 (4.43)		Not identified	
Arizona and New York	Watershed areas (*n* = 9)	Eleven months	Surface water (*n* = 1053)	*Listeria* spp. (excludes *Lm*)	Yes	AZ: 0/76 (0) NY: 58/257 (22.57)	Streams in NY	*L. booriae, L. innocua, L. marthii, L. seeligeri,* and *L. welshimeri*	[43]
				*Listeria monocytogenes*	Yes	AZ: 3/76 (3.95) NY: 30/257 (11.67)	Canals in AZ and streams in NY	Not identified	
Arizona	Five sites in two wastewater treatment plants	20 months	Reclaimed and return flow water (*n* = 28)	*Listeria monocytogenes*	ND	N/A	N/A	N/A	[44]
Mid-Atlantic U.S.	Convention water sources (*n* = 6)	Three years	Tidal freshwater river (*n* = 34), non-tidal freshwater creek (*n* = 32), reclaimed water holding pond (*n* = 25), pond water sites (*n* = 69), and produce wash water (*n* = 10)	*Listeria monocytogenes*	Yes	53/170 (31.18)	All sample types	Not identified	[45]
Washington	Red raspberry field with individual plots (22.86 m × 3.05 m; *n* = 4) with buffer rows between treatment plots; completed in duplicate	Two years	Fertilizer, soil, foliar, and raspberry fruit	*Listeria monocytogenes*	ND	N/A	N/A	N/A	[46]
New York	Produce farms (*n* = 5)	27 months	Soil, water (engineered and surface), feces, and drag swabs (*n* = 588)	*Listeria monocytogenes*	Yes	88/588 (15.0)	All sample types except for engineered water	Not identified	[47]
New York	Produce farms (*n* = 21)	Five weeks	Fields (*n* = 263) and environmental samples (soil, drag swab, and water; *n* = 600)	*Listeria monocytogenes*	Yes	Field: 46/263 (17.5) Soil: 30/263 (11) Drag: 21/263 (8) Water: 22/74 (30)	All sample types	Nine allelic types representing lineages I, II, and IIIa	[48]
New York	Produce farms (*n* = 10)	Six weeks	Terrestrial, water, and fecal (*n* = 124)	*Listeria* spp. (excludes *Lm*)	Yes	24/124 (19.35)	All sample types	*L. seeligeri, L. welshimeri,* and *L. innocua*	[49]
				*Listeria monocytogenes*	Yes	28/124 (22.58)	All sample types	Not identified	
**Packinghouse, Indoor Production, and Processing**									
Pennsylvania	Small scale mushroom production facility (*n* = 1)	Two months	NFCS, such as shovels, drains, doors, floors, conveyor belts, brooms, dust pans, etc. (*n* = 184)	*Listeria* spp. (excludes *Lm*)	Yes	26/184 (14.13)	Phase I composting, phase II composting, tray filling line, and growing rooms	*L. innocua, L. welshimeri,* and *L. grayi*	[50]
				*Listeria monocytogenes*	Yes	3/184 (1.63)	Phase I composting	Not identified	
Maryland	Organic farms (*n* = 7)	Two years	Produce (tomatoes, leafy greens, peppers, cucumbers, etc.), well water, and surface water (*n* = 206)	*Listeria monocytogenes*	ND	N/A	N/A	N/A	[51]
Southern U.S.	Farms (*n* = 13) and packing sheds (*n* = 5)	19 months	Produce (leafy greens, herbs, and cantaloupe; *n* = 398)	*Listeria monocytogenes*	ND	N/A	N/A	N/A	[52]
Northeast U.S.	Apple and other tree fruit packinghouses (*n* = 3)	Six months	NFCS (*n* = 117)	*Listeria monocytogenes*	Yes	66/117 (56.41)	Washing, drying, and waxing areas of all facilities	Not identified	[53]
Southern U.S.	Packinghouses (*n* = 8)	14 months	Leafy greens (*n* = 109), herbs (*n* = 165), and cantaloupe (*n* = 36)	*Listeria monocytogenes*	Yes	Leafy greens: 3/43 (6.98)	Cabbage	Not identified	[54]
Southeastern U.S.	Packinghouses (*n* = 11)	Nine months	NFCS, such as forklift wheels, drains, dump tank legs, cold room floors, etc.	*Listeria* spp. (excludes *Lm*)	Yes	52/1588 (3.27)	Drains, cold storage rooms, wet NFCS, mobile NFCS, dry NFCS, and outside packing/handling area	*L. innouca, L. marthii, L. seeligeri,* and *L. welshimeri*	[55]
				*Listeria monocytogenes*	Yes	60/1588 (3.78)		Not identified	
California	Stone fruits (*n* = 105; from seven lots)	Not specified	White nectarines (*n* = 30), yellow nectarines (*n* = 30), white peaches (*n* = 30), and yellow peaches (*n* = 15)	*Listeria monocytogenes*	Yes; 11.3 CFU/fruit (geometric mean)	Total: 56/105 (53.3) Nectarines: 15/60 (25) Peaches: 41/45 (91.1)	All sample types	IVb-v1 and 1/2b	[17]
Four U.S. states	Packinghouses (*n* = 3) and fresh-cut facilities (*n* = 5)	One year	Sponge samples (*n* = 2014)	*Listeria* spp. (excludes *Lm*)	Yes	Packinghouse: 5/252 (2) to 8/171 (4.7) Fresh-cut: 0/249 (0) to 5/325 (1.5)	Zones 2 and 3 for packinghouse and only zone 3 for fresh-cut	Not identified	[56]
				*Listeria monocytogenes*		Packinghouse: 2/252 (0.8) to 10/171 (5.8) Fresh-cut: 0/249 (0) to 4/246 (1.6)	Zones 2 and 3 for packinghouse and only zone 3 for fresh-cut	Not identified	
Multiregional	Processing plants (*n* = 2)	14 months	Baby spinach (*n* = 409)	*Listeria* spp. (excludes *Lm*)	Yes	2/409 (0.49)	Processed samples	*L. seeligeri*	[57]
				*Listeria monocytogenes*	Yes	3/409 (0.73)	One processed and two minimally processed baby spinach samples	Not identified	
Pacific Northwest U.S.	Produce handling and processing facilities (*n* = 7)	One year	Environmental sponge samples (*n* = 350)	*Listeria* spp. (excludes *Lm*)	Yes	11/350 (3.14)	Drain, entry point, floor, forklift tire, forklift traffic area, equipment leg	*L. innocua, L. ivanoii,* and *L. welshimeri*	[58]
				*Listeria monocytogenes*	Yes	15/350 (4.29)		1/2a, 3a, 4b, 4d, 4e	
California	Grower (*n* = 1)	Four months	Conventional and organic spring mix	*Listeria monocytogenes*	ND	N/A	N/A	N/A	[59]
Not specified	Fresh mushroom slicing and packaging operation; 98 sampling sites within the facility	14 months	NFCS, such as loading dock doors, floors, walls, pallets, drains, squeegees, electrical utility boxes, forklifts, plastic curtains, etc. (*n* = 255)	*Listeria* spp. (excludes *Lm*)	Yes	16/255 (6.27)	Receiving and staging, washing and slicing, packaging, and shipping sites	*L. innocua* and *L. grayi*	[60]
				*Listeria monocytogenes*	Yes	48/255 (18.8)	Receiving and staging, washing and slicing, and packaging sites	1/2a, 1/2b, and 1/2c	
**Retail**									
Michigan and New Jersey	Distribution (cilantro), retail (cilantro and mung bean sprouts) and farm (cucumber)	Not specified	Cilantro (pre-retail and retail), cucumbers, and mung bean sprouts	*Listeria* spp. and *Listeria monocytogenes*	No live isolates obtained; however, species level proportional abundances illustrate presence of *Listeria* DNA		*L. monocytogenes* DNA present in two cilantro samples	Not identified	[61]
South America, North America, Europe, Africa, and Asia	Published studies (*n* = 25)	Not specified	Packaged salads (*n* = 20,904), including packaged greens (*n* = 1212), packaged RTE (*n* = 11,978), unsure if packaged (*n* = 2637), and packaged with meat (*n* = 5077)	*Listeria monocytogenes*	Yes	543/20,904 (2.60)	All sample types, except for some unsure if packaged samples	Not identified	[62]
Maryland	Retail stores (*n* = 3)	One year	Basil, cilantro, lettuce, scallion, spinach, and parsley (*n* = 414)	*Listeria monocytogenes*	Yes	Not specified	Spinach	Not identified	[63]
California, Texas, Iowa, Minnesota, Ohio, Massachusetts, and Florida	Retail grocery produce departments (*n* = 30)	Eight months	FCS and NFCS	*Listeria monocytogenes*	Yes	Total: 226/5112 (4.42) NFCS: 178/2205 (8.1) FCS: 48/2907 (1.7)	Drain (cold room storage), standing water, drain (produce area), squeegee/floor cleaners, floor (cold room storage), etc.	Not identified	[64]
Maryland and California	Retail markets	Over 14 to 23 months	Bagged salads (*n* = 2966)	*Listeria monocytogenes*	Yes	Total: 22/2966 (0.74) MD: 8/1465 (0.55) CA: 14/1501 (0.93)	Bagged salads	Not identified	[65]
California, Maryland, Connecticut, and Georgia	Retail stores (*n* = 1042)	Two years	Produce, including cut vegetables (raw), low-acid cut fruit, and sprouts (*n* = 6749)	*Listeria monocytogenes*	Yes	36/6749 (0.53)	All sample types	Not identified	[66]
Virginia	Retail markets	Two months	Whole (*n* = 20) and sliced (*n* = 8) shiitake mushrooms	*Listeria* spp. (excludes *Lm*)	Yes	Total: 3/28 (10.71) Whole: 1/20 (5) Sliced: 2/8 (25)	Whole and sliced mushrooms	Not identified	[67]
				*Listeria monocytogenes*	ND	N/A	N/A	N/A	
Delaware	Grocery stores (number not specified)	15 months	Mushrooms (*n* = 202) and alfalfa sprouts (*n* = 206)	*Listeria* spp. (excludes *Lm*)	Yes	Total: 24/408 (5.88) Mushroom: 17/202 (8.42) Sprouts: 7/206 (3.40)	Mushroom and sprouts	*L. welshimeri, L. innocua,* and *L. seeligeri*	[68]
				*Listeria monocytogenes*		Total: 1/408 (0.25) Mushroom: 0/202 Sprouts: 1/206 (0.49)	Only sprouts	Not identified	
Seattle, Washington	Retail stores	One year	Sprouts (*n* = 200) and mushrooms (*n* = 100)	*Listeria monocytogenes*	Yes	1/100 (1)	Mushroom	Not identified	[69]
Colorado, Connecticut, Georgia, Maryland, Minnesota, California, Texas, and Washington	Retail locations	Six years	Leafy greens (*n* = 14,183), sprouts (2652), and melons (3411)	*Listeria monocytogenes*	Yes	Leafy greens: (0.11) Sprouts: (0.11) Melons: (0.23)	Spinach, romaine, alfalfa sprouts, broccoli sprouts, cucumber, and mango	Not identified	[70]
Philadelphia, Pennsylvania	Retail food establishments (*n* = 60)	Two years	RTE fresh fruit, greens, and herbs	*Listeria monocytogenes*	ND	N/A	N/A	N/A	[71]
Minnesota	Supermarket	Not specified	Produce (lettuce, potato peels, corn husks, broccoli stems, cabbage outer leaves, carrot peels, cauliflower stems, mushroom stems, spinach, beet peels, and frozen green beans, pea pods, green peas, and spinach)	*Listeria monocytogenes*	ND	N/A	N/A	N/A	[72]
Minneapolis, Minnesota	Supermarkets (*n* = 2)	One year	Produce (broccoli, cabbage, carrots, cauliflower, cucumbers, lettuce, mushrooms, potatoes, radishes, and tomatoes; *n* = 1000)	*Listeria* spp. and *Listeria monocytogenes*	Yes	97/1000 (9.7)	Lettuce, cabbage, cucumbers, mushrooms, potatoes, radishes	*L. monocytogenes, L. innocua, L. welshimeri,* and *L. seeligeri*	[73]
**Farmers’ Markets**									
West Virginia and Kentucky	Farmers’ markets (*n* = 2)	Four months	Produce (tomatoes, peppers, cucumber, cantaloupe, and spinach; *n* = 212)	*Listeria* spp. (excludes *Lm*)	Yes	4/212 (1.89)	Peppers and cantaloupes	Not identified	[74]
				*Listeria monocytogenes*	Yes	4/212 (1.89)	Tomatoes, cucumbers, and cantaloupes	Not identified	
Pennsylvania	Farmers’ markets (*n* = 25) and vendors (*n* = 58)	8 months	Leafy greens (*n* = 50 each of lettuce, spinach, and kale)	*Listeria* spp. (excludes *Lm*)	Yes	5/152 (3.30)	Kale, lettuce, and spinach	Not identified	[75]
				*Listeria monocytogenes*	Yes	1/152 (0.66)	Spinach		
Washington D.C.	Farmers’ markets and supermarkets	Not specified	Produce (alfalfa sprouts, beets, broccoli, broccoli sprouts, cauliflower, celery, cilantro, cucumbers, field cress, green peppers, lettuce, mung bean sprouts, potatoes, soybean sprouts, watercress, yams; *n* = 127)	*Listeria* spp. (excludes *Lm*)	Yes	19/127 (14.96)	Celery, field cress, lettuce, mung bean sprouts, potatoes, soybean sprouts, watercress, yams	*L. innocua, L. welshimeri,* and *L. grayi*	[76]
				*Listeria monocytogenes*	Yes	6/127 (4.72)	Field cress and potatoes	N/A	
Florida	Farmers’ markets (*n* = 9) and supermarkets (*n* = 12)	10 months	Leafy greens (*n* = 103), berries (*n* = 106), spinach (*n* = 77), and tomatoes (*n* = 115)	*Listeria monocytogenes*	Yes	4/401 (1)	Leafy greens and spinach from farmer’s markets	Not identified	[77]
**Domestic**									
Philadelphia, Pennsylvania	Homes (*n* = 100)	One year	Refrigerator door handle, bottom shelf, meat drawer; kitchen counter near sink; used kitchen sponge or dishcloth	*Listeria* spp. (excludes *Lm*)	Yes	12/557 (2.15)	Meat drawer	*L. innocua, L. welshimeri, L. grayi,* and *L. seeligeri*	[78]
				*Listeria monocytogenes*	Yes	4/557 (0.72)	Refrigerator door handle, refrigerator drawer, kitchen sink, and dishcloth/sponge	Not identified	

**Table 3:** Range of *Listeria* spp. and *Lm* prevalence and identified *Listeria* spp. and *Lm* serovars by supply chain stage.

Stage of Supply Chain	*Listeria* spp. Prevalence (%) ^a,b^		Identified *Listeria* Species	*Lm* Prevalence (%)		Identified *Lm* Serovars ^c^
	Low	High		Low	High	
Natural environment and outdoor production	ND	46.81 [28]	*L. welshimeri, L. innocua, L. seeligeri, L. grayi, L. ivanoii, L. marthii, L. booriae*	ND	61.1 [31]	1/2a, 1/2b, 3a, 3b, 4d, 4e, 7
Packinghouse, indoor production, and processing	0.08	14.13 [50]	*L. welshimeri, L. innocua, L. seeligeri, L. grayi, L. ivanoii, L. marthii*	ND	56.41 [53]	1/2a, 1/2b, 1/2c, 3a, 4b, 4c, 4d, IVb-v1
Retail	6.13 [68]	10.71 [67]	*L. innocua, L. welshimeri, L. seeligeri*	ND	4.42 [64]	NI
Farmers’ markets	ND	14.96 [76]	*L. welshimeri, L. innocua, L. seeligeri, L. grayi*	ND	4.72 [76]	NI
Domestic	2.15 [78]		*L. welshimeri, L. innocua, L. seeligeri, L. grayi*	0.72 [78]		NI

^a^*Listeria* spp. exclude *Lm* except for the high *Listeria* spp. prevalence at the natural environment and outdoor production stage which includes *Lm.* ^b^ Not detected (ND) ^c^ Not identified (NI).

## Data Availability

No new data were created or analyzed in this study. Data sharing is not applicable to this article.
